# Successful rapid desensitization to multiple insulin preparations in an adult with type 1 diabetes: a case report and literature review

**DOI:** 10.3389/fendo.2026.1715012

**Published:** 2026-01-16

**Authors:** Yuwen Chen, Wenwen Zhu, Guoping Chen, Jianping Yao

**Affiliations:** 1 Department of Endocrinology, Huzhou Central Hospital, Fifth School of Clinical Medicine of Zhejiang Chinese Medical University, Affiliated Central Hospital of Huzhou University, Huzhou, Zhejiang, China; 2 Department of Endocrinology, Deqing People's Hospital, Huzhou, Zhejiang, China

**Keywords:** case report, continuous subcutaneous insulin infusion, desensitization, insulin allergy, type1 diabetes

## Abstract

**Background:**

Insulin allergy, although rare in type 1 diabetes (T1DM), poses a significant clinical challenge due to the indispensable role of insulin therapy. Rapid induction of insulin tolerance is critical for affected individuals, especially in acute complications such as diabetic ketoacidosis (DKA).

**Case presentation:**

We report a case of a 50-year-old male with newly diagnosed T1DM who developed type I hypersensitivity reactions to multiple insulin analogs, manifesting as localized erythema, pruritus, and induration. After conventional management, including switching insulin preparations, proved ineffective, a rapid desensitization protocol was initiated using continuous subcutaneous insulin infusion (CSII). Preceding the pump initiation, half of the estimated basal dose of insulin glargine was administered subcutaneously. CSII with insulin aspart was then started at an extremely low initial rate, with increments every 30 minutes.

**Results:**

The target basal infusion rate was successfully achieved within 5 hours without the use of antihistamines or corticosteroids. The procedure was well-tolerated, with no systemic or local allergic reactions. Following desensitization, the patient successfully transitioned to daily injections of glargine and pre-meal aspart insulin, with no recurrence of allergic reactions during long-term follow-up.

**Conclusion:**

A CSII-based rapid desensitization protocol is a safe, effective, and efficient strategy for managing insulin allergy in T1DM, including cases with sensitivities to multiple insulin preparations. This approach is particularly suitable for patients requiring urgent insulin therapy.

## 1 Introduction

Insulin allergy, though uncommon since the advent of recombinant human insulin (estimated prevalence ~2%) (1), remains a serious clinical concern. It can manifest as type I (IgE-mediated), type III (immune complex-mediated), or type IV (T-cell-mediated) hypersensitivity, ranging from local cutaneous reactions to systemic anaphylaxis (2). Rapid induction of insulin tolerance is critical, especially in acute complications such as diabetic ketoacidosis (DKA).

Traditional desensitization protocols are often prolonged, require adjunctive anti-allergy medications, and may mask true allergic responses. In recent years, CSII has emerged as a valuable tool for insulin desensitization due to its ability to deliver precise micro-doses and facilitate gradual increments (3, 4). We present a case of successful multi-insulin desensitization using CSII within 5 hours, offering a novel and efficient therapeutic approach.

## 2 Case report

**Figure 1:**
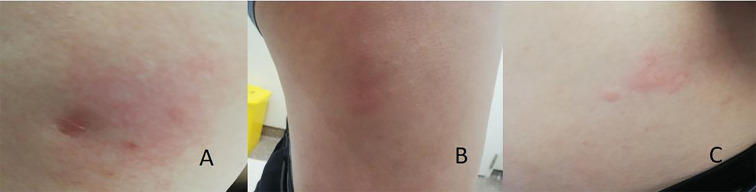
Immediate skin reactions to insulin injections: **(A)** periumbilical lispro; **(B)** right abdominal glargine; **(C)** left abdominal aspart.

A 50-year-old male was diagnosed with type 1 diabetes (T1DM) and DKA in March 2022. Initial therapy included premixed aspart insulin 30 (NovoMix^®^ 30, tid) and acarbose (used to mitigate postprandial hyperglycemia during allergy evaluation), with acceptable glycemic control. Ten days later, he developed pruritus, erythema, and induration at injection sites. Despite switching to various insulin formulations (Novolin 30R, aspart, Insulin degludec and aspart, glargine U-300, lispro) and even needle-free injectors, local reactions persisted. He was re-admitted in November 2022. Physical examination revealed BP 140/82 mmHg, BMI 25.3 kg/m², and erythematous nodules at injection sites. Lab results included fasting glucose 7.91 mmol/L, C-peptide <0.01 nmol/L, HbA1c 7.9%, and total IgE 1038 IU/mL (ref: <87 IU/mL). Intradermal testing was conducted using different insulin formulations (dilution ratio 1:10). Skin testing showed immediate positive reactions to aspart, glargine and lispro (**Figure 1**). Within 15 minutes of insulin injection, localized erythematous, pruritic wheals appeared on the skin, lasting no longer than 48 hours.

A diagnosis of T1DM with type I insulin allergy was established. A CSII-based rapid desensitization protocol was initiated. No premedication with antihistamines or corticosteroids was administered. The desensitization was conducted in a specialized endocrinology ward equipped with resuscitation equipment and epinephrine. Continuous electrocardiogram (ECG) monitoring was performed, with vital signs assessed every 15 minutes and capillary glucose every 30 minutes. The procedure was to be halted for any systemic reaction or severe local progression. When patients exhibit pronounced thirst, frequent urination, gastrointestinal discomfort such as nausea, vomiting, and abdominal pain, deep breathing with breath smelling like rotten apples, or blood glucose levels exceeding 13.9 mmol/L, immediate testing for blood gases, blood ketones, or urinary ketones is required. The test should be discontinued if necessary, and treatment with fluid replacement and insulin should be initiated. At 4 AM, 10 U of glargine U-300 (50% of estimated basal dose) was injected subcutaneously in the left abdomen, eliciting a local reaction. At 8 AM, CSII was started with aspart insulin diluted 1:10,000, beginning at 0.000025 U/h. The rate was increased every 30 minutes under close glucose and allergy monitoring (**Table 1**). The patient remained fasting, with oral glucose given for hypoglycemia. After 5 hours, the rate reached 2.4 U/h without allergic reaction.

**Table 1:** Insulin desensitization protocol.

Minutes	Pump basal rate (mL/h)	Final concentration (U/mL)	Insulin delivery rate (U/h)	Capillary blood glucose (mmol/L)	Comments	Notes
0	0.25	0.0001	0.000025	8.9		Add 1000 ml NS to 1U, withdraw 1 ml, then add 9 ml NS
30	0.1	0.001	0.0001	7.0		1U plus 1000ml NS
60	1	0.001	0.001	6.2		1U plus 1000ml NS
90	2	0.001	0.002	5.3	10g glucose	1U plus 1000ml NS
120	2	0.002	0.004	6.7		1U plus 500ml NS
150	2	0.004	0.008	7.0		1U plus 250ml NS
180	2	0.008	0.016	5.3		2U plus 250ml NS
210	2	0.016	0.032	5.5		4U plus 250ml NS
240	0.06	1	0.06	5.8		Undiluted
270	1.20	1	1.20	5.3	10g glucose	Undiluted
300	2.4	1	2.4	5.2	10g glucose	Undiluted^1^

^1^The patient remained fasting, with oral glucose given for hypoglycemia.

**Figure 2:**
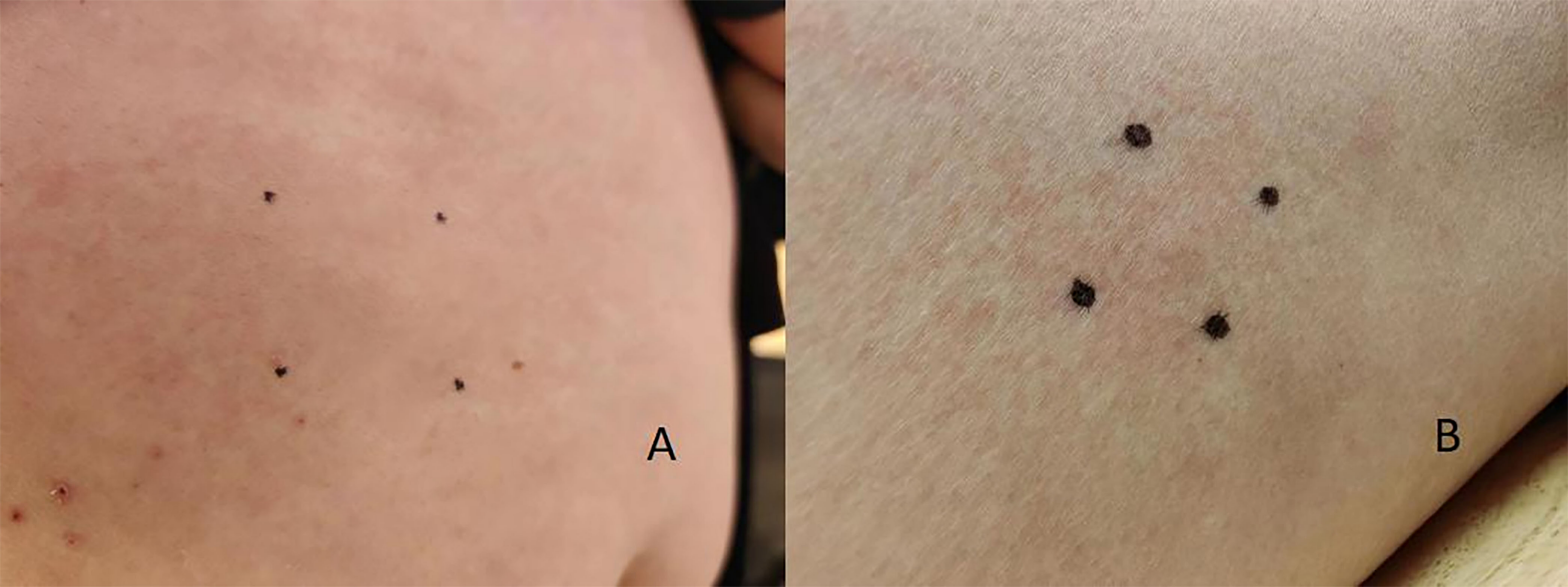
Post-desensitization: **(A)** aspart injection site; **(B)** glargine injection site.

5 hours later, insulin pump therapy with aspart was continued. Simultaneously, glargine was initiated at a starting dose of 2 units administered subcutaneously, with increments of 2 units every 2 hours. Concurrently, the basal rate of insulin aspart was reduced. When glargine was increased to 6 units, no allergic reaction occurred. Subsequently, the aspart insulin pump was discontinued. Treatment was switched to daily injections of 18 units of glargine and pre-meal injections of aspart. No adverse skin reactions were observed during this period (**Figure 2**).

**Figure 3:**
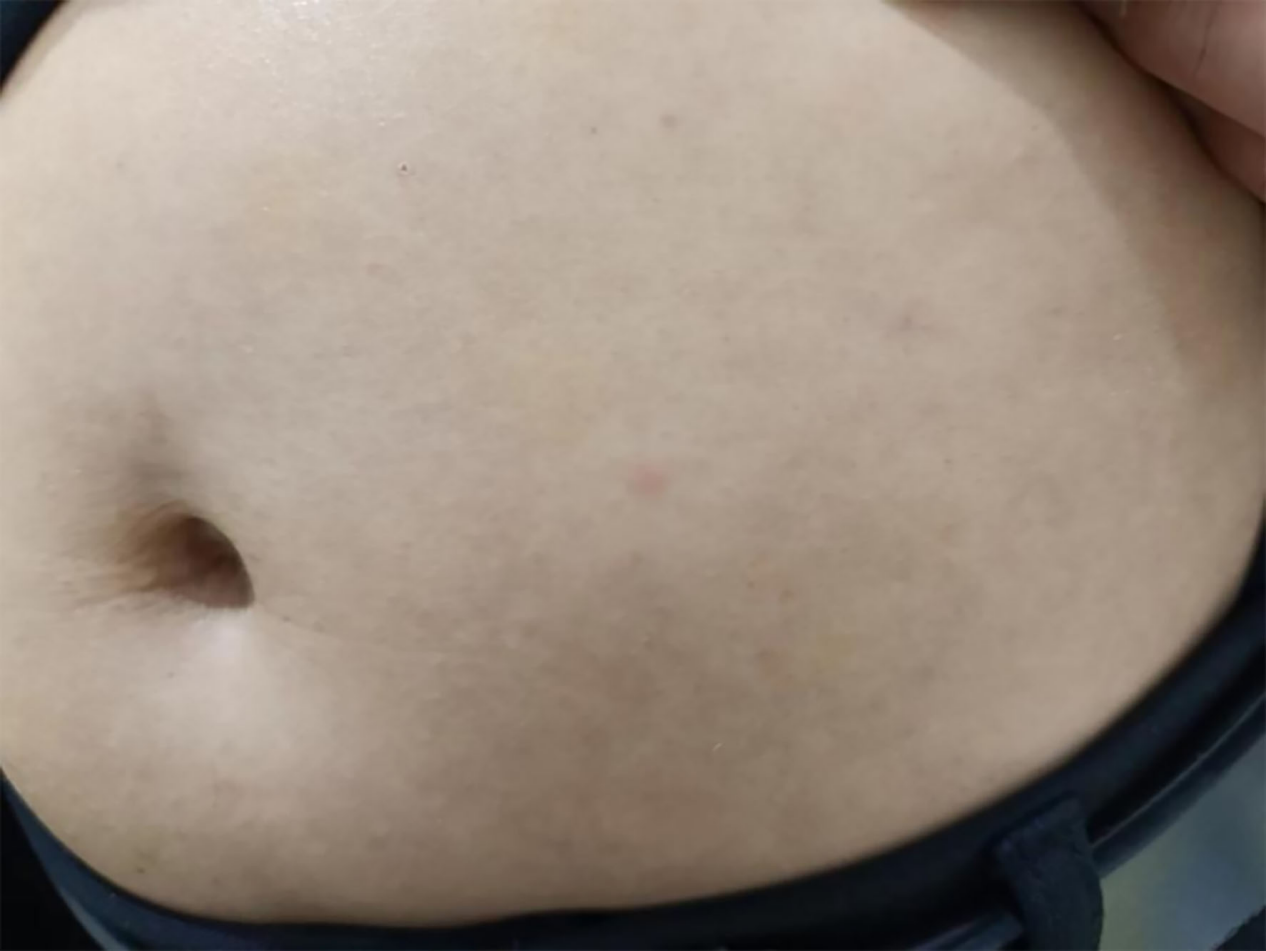
No allergic reactions during follow-up with glargine and aspart injections.

During follow-up until April 2023, no skin reactions were observed (**Figure 3**). Notably, although glargine was not formally desensitized via CSII, it was well-tolerated when reintroduced at low doses and titrated to the target without allergic recurrence.

## 3 Discussion

Insulin allergy is a rare yet complex complication of insulin therapy in diabetic patients. Since the introduction of recombinant human insulin, the incidence of insulin allergic reactions has decreased significantly, occurring in approximately 2% of patients. Of these cases, less than one-third are considered insulin-related, while the majority are attributed to excipients present in insulin preparations, such as zinc, protamine, and metacresol (1). The patient developed desensitization to aspart insulin through continuous subcutaneous insulin infusion(CSII). However, when glargine insulin was reintroduced at a low dose without undergoing a structured desensitization protocol, the patient did not exhibit an allergic reaction to glargine insulin. Therefore, we concluded that the patient’s allergic reactions to multiple insulin preparations was induced by insulin itself. While the patient’s reactions to multiple insulin analogs suggest sensitivity to the insulin molecule itself, we acknowledge that allergy to excipients (e.g., metacresol, zinc) cannot be fully ruled out in the absence of specific excipient skin testing or *in vitro* assays.

Given the essential role of insulin in the management of type 1 diabetes, insulin desensitization remains the treatment of choice for affected individuals. Reported approaches include antihistamines, corticosteroids, addition of glucocorticoids to insulin, switching to recombinant human insulin or analogs, gradual dose escalation, continuous subcutaneous insulin infusion (CSII) (5), and intravenous insulin combined with CSII (6). A summary of reported cases of insulin desensitization in T1DM over the past twenty-five years is provided in **Table 2**.

**Table 2:** A summary of reported cases of insulin desensitization in T1DM over the past twenty-five years.

References	Gender	Age(years)	Types of insulin	Presenting symptoms	Hypersensitivity reaction type	Protocol	Duration of protocol
Eapen et al., 2000 (7)	Female	5	Regular insulin, NPH	Swelling and pain at the injection site, accompanied by rash and hives	Not reported	CSII	8h
Näf et al., 2002 (8)	Male	43	Regular insuli,NPH,Lispro	Itching, redness, and swelling at the injection site; Generalized urticaria	Not reported	CSII	22h
Sola-Gazagnes et al., 2003 (9)	Female	21	Animal or human insulin, NPH, Regular insulin	Localized urticaria	Type I	CSII	Not reported
Matheu et al., 2005 (10)	Male	25	Aspart, Lispro, Regular insulin, NPH	wheals, Pruritus, dyspnea, subcutaneous nodules	Type I, Type III	CSII	16d
Neville et al., 2008 (6)	Male	9(2 cases)	Regular insulin, NPH	Generalized urticaria and lip swelling	Type I	Intravenous insulin infusion combined with CSII	5-6d
Koroscil et al., 2011 (11)	Female	18	Aspart, Regular insulin, Glulisine, Lispro, Glargine, NPH	Localized itching and erythema; Firm and tender nodules	Type I, Type III	Pancreatic transplantation was performed following failure of insulin desensitization with intravenous insulin infusion and CSII	
Wheeler et al., 2012 (12)	Female	12	Metacresol	Localized erythema with pain	Type I, Type IV	Intravenous insulin infusion combined with CSII	312h
Hasselmann et al., 2013 (5)	Male	8	Protamine, Glargine, Lispro	Pruritic wheals; Generalized urticaria	Not reported	CSII	36h
Rojas et al., 2014 (13)	Male	17	NPH, Lispro	Angioedema, wheezing, shortness of breath, and pruritic rash	Type I	Frequent subcutaneous administration of small, incremental doses of glargine	5d
Tian et al., 2021 (14)	Female	40	Aspart, Glargine U-100, Glulisine, Detemir, Lispro protamine/Lispro mix	Pruritic wheals	Type I	CSII	5h
He et al., 2022 (15)	Male	34	Novolin R, Novolin N	Pruritus, wheals, and subcutaneous induration	Not reported	Frequent subcutaneous administration of small, incremental doses of Novolin R and insulin detemir	3d
Alkhatib et al., 2023 (2)	Female	18	Lispro, Glargine, Detemir	Respiratory distress; Generalized urticaria	Type I, Type IV	Pancreatic transplantation was performed following failure of insulin desensitization with intravenous insulin infusion and CSII	
Alkhatib et al., 2023 (2)	Female	16	Aspart, Lispro, Glulisine, Glargine, Detemir, NPH, Regular insulin	Bruising, erythema and leg pain	Type III	Pancreatic transplantation was performed following failure of insulin desensitization with intravenous insulin infusion and CSII	
Alkhatib et al., 2023 (2)	Male	8	Aspart, Lispro, Degludec, Glargine, Regular insulin	Eczematous and erythematou plaques	Type IV	Failure of insulin desensitization in CSII	
Alkhatib et al., 2023 (2)	Male	15	Aspart, Glulisine	Erythematous, firm and tender nodules	Type III, Type IV	Failure of insulin desensitization in CSII	

As summarized in **Table 2**, insulin desensitization has been documented in patients across a wide age range with varying clinical manifestations. The duration of desensitization in reported cases varies significantly, from several hours to multiple days, with some even requiring pancreatic transplantation in highly refractory cases. Compared to these previous reports, our case stands out for its remarkably rapid and efficient protocol: achieving successful desensitization within just 5 hours using CSII alone, without the need for adjunctive antihistamines or corticosteroids. This highlights a notable advantage in terms of both time efficiency and procedural simplicity, offering a particularly viable option for urgent clinical scenarios such as diabetic ketoacidosis (DKA).

Desensitization protocols typically involve frequent, small, and progressively increasing doses of insulin. CSII offers an ideal delivery method for this process, avoiding repeated injections, enabling ultra-low starting doses, allowing flexible rate adjustments, and improving patient compliance (4).

This report presents a case of successful rapid desensitization to multiple insulin analogs using CSII in an adult with T1DM. Since the initial micro-doses delivered by CSII are insufficient to meet metabolic needs—potentially leading to hyperglycemia and ketosis—50% of the estimated basal insulin dose (glargine U-300) was administered subcutaneously before initiating the pump. The patient remained fasting throughout the procedure to minimize glycemic excursions, with close glucose monitoring and carbohydrate supplementation as needed to prevent hypoglycemia.

The mechanism underlying CSII-induced insulin desensitization is not fully elucidated. It has been proposed that CSII may be effective due to the minimal depot formation at the infusion site (16), potentially leading to sustained mast cell degranulation and subsequent blockade of allergic responses (17). Other hypotheses include induction of T-cell anergy or exhaustion, activation of regulatory T cells, and modulation of antibody responses. Desensitization may also involve a decrease in insulin-specific IgE and a gradual increase in IgG antibodies (18, 19).

Notably, in this case, although formal desensitization via CSII was performed only with aspart insulin, the patient subsequently tolerated glargine without a structured protocol. The glargine dose was cautiously reintroduced at a low dose and gradually titrated to the target level without recurrence of allergic reactions. This observation may indicate potential immunologic cross-tolerance between insulin analogs (20), although a formal desensitization effect from the gradual, low-dose reintroduction of glargine cannot be excluded. Similar phenomena have been reported in other cases where successful desensitization to one insulin preparation resulted in broad tolerance to others, eliminating the need for multiple drug-specific procedures (20, 21).

This case demonstrates that CSII enables rapid insulin desensitization within hours. This approach overcomes the clinical challenges of prolonged desensitization periods and glycemic instability, making it particularly suitable for emergency situations such as DKA. Moreover, no antihistamines or corticosteroids were used during the process, avoiding potential masking of true allergic reactions and suggesting that adjuvant anti-allergy medications may not always be necessary.

## 4 Conclusion

CSII-based rapid desensitization is a safe, effective, and efficient strategy for managing insulin allergy in T1DM, including cases with multiple insulin sensitivities. The induction of cross-tolerance to non-desensitized insulin analogs merits further investigation. Larger studies are needed to validate this protocol and elucidate its immunologic mechanisms.

## Data Availability

The original contributions presented in the study are included in the article/supplementary material. Further inquiries can be directed to the corresponding authors.
